# Updating the Knowledge on the Secretory Machinery of Hops (*Humulus lupulus* L., Cannabaceae)

**DOI:** 10.3390/plants13060864

**Published:** 2024-03-17

**Authors:** Felipe Paulino Ramos, Lucas Iwamoto, Vítor Hélio Piva, Simone Pádua Teixeira

**Affiliations:** 1 Departamento de Ciências Farmacêuticas, Faculdade de Ciências Farmacêuticas de Ribeirão Preto (FCFRP), Universidade de São Paulo (USP), Ribeirão Preto 14040-903, Brazil; felipepramos@usp.br (F.P.R.); lucas.iwamoto@usp.br (L.I.); vitor.piva@usp.br (V.H.P.); 2 Departamento de Biologia, Faculdade de Filosofia, Ciências e Letras de Ribeirão Preto (FFCLRP), Universidade de São Paulo (USP), Ribeirão Preto 14040-901, Brazil

**Keywords:** anatomy, glands, secretory idioblasts, latex, laticifer, ultrastructure

## Abstract

Cannabaceae species garner attention in plant research due to their diverse secretory structures and pharmacological potential associated with the production of secondary metabolites. This study aims to update our understanding of the secretory system in Hops (*Humulus lupulus* L.), an economically important species especially known for its usage in beer production. For that, stems, leaves, roots, and inflorescences were collected and processed for external morphology, anatomical, histochemical, ultrastructural and cytochemical analyses of the secretory sites. Our findings reveal three types of secretory structures comprising the secretory machinery of Hops: laticifer, phenolic idioblasts and glandular trichomes. The laticifer system is articulated, anastomosing and unbranched, traversing all plant organs, except the roots. Phenolic idioblasts are widely dispersed throughout the leaves, roots and floral parts of the species. Glandular trichomes appear as two distinct morphological types: capitate (spherical head) and peltate (radial head) and are found mainly in foliar and floral parts. The often-mixed chemical composition in the secretory sites serves to shield the plant from excessive UVB radiation, elevated temperatures, and damage inflicted by herbivorous animals or pathogenic microorganisms. Besides the exudate from peltate glandular trichomes (lupulin glands), latex and idioblast content are also likely contributors to the pharmacological properties of different Hop varieties, given their extensive presence in the plant body.

## 1. Introduction

Secretory structures in plants consist of individualized cells or multicellular structures that produce, accumulate, and/or release secondary metabolites. These structures exhibit significant morphological and chemical diversity, playing a crucial role in the plant’s interaction with its environment [1]. The family Cannabaceae, particularly economically important species like *Humulus lupulus* L. (Hop) and *Cannabis sativa* L. (hemp), have long captured the attention of researchers due to the ability of their secretory structures to produce chemical substances with therapeutic value [2,3,4,5,6,7].

*Humulus lupulus*, known for imparting a bitter taste and characteristic aroma to beer, owes these attributes to chemical compounds such as *α*-acids, primarily found in the bracts of pistillate flowers [8,9]. Beer has a global production and consumption history, dating back to ancient times when introduced by early civilizations [10]. *H. lupulus* also houses numerous bioactive compounds with established pharmacological potential and medicinal value. Research indicates its effectiveness in preventing and treating various modern-day disorders [11]. Recent reviews comprehensively synthesize knowledge on the chemical and pharmacological properties of hundreds of phytochemicals and secondary metabolites in this species [12], emphasizing its polyphenolic profile [13]. Its pistillate inflorescences, also called dried “strobiles” or “cones”, contain bitter substances used as sedative agents in folk medicine. These substances address conditions like nervous tension, headaches, sleep disorders [14,15,16], and also reportedly help with lack of appetite, anemia, bacterial infections, dermatitis, and diarrhea [17]. In recent years, Hop extracts have shown efficacy in reducing oxidative stress induced by iron overload [18] and exhibit anti-coronavirus properties [19], demonstrating diverse applications in the biomedical field. Reports from the past century highlight the diverse traditional uses of *H. lupulus*, extending beyond medicinal applications. Inflorescences are used for various purposes, such as producing food oil [20], and in manufacturing perfumes, deodorants, and skin lotions [21]. They also play a role in bread making and yeast cultivation [22]. Stems of *H. lupulus* provide rope fibers and cattle bedding in the UK [20], and contribute to cloth production in Sweden [22]. In the Mediterranean region and Western Europe, young leaves of the plant are consumed as vegetables [21,22,23]. Despite the lack of vibrant colors in its small flowers, records show the entire plant being used for ornamental purposes [22].

Given the extensive knowledge about the identification and biological activities of chemical compounds, information about the sites where these compounds are produced in *H. lupulus* remains limited. Similar to *C. sativa* [2,24,25], Hop is known to possess a laticifer system, tannin idioblasts, and glandular trichomes. However, detailed studies on these secretory sites, especially the first two types, are lacking for the species. The report of latex ducts in the root cortex of *H. lupulus* [26] was refuted [27,28]. More recently, a non-articulated and unbranched laticifer system was illustrated as a photomicrograph [24], without further details. Finally, a formal examination of laticifer system anatomy and distribution was conducted [29]. Peltate glandular trichomes, also known as lupulin glands, have received significantly more attention than the laticifer system in this species. However, existing studies primarily focus on proteomics and the description of biochemical pathways of secondary metabolites [30,31,32,33,34], with few records of histochemical tests and ultrastructural analyses [35,36,37,38,39]. Much like in the case of the laticifer system, there are very few records of the presence of secretory idioblasts in *H. lupulus*. Two articles from the 1950s [28,40] on the morpho-anatomy of the Hop root feature figures and analyses of its ‘tannin- and resin-containing cells’. Remarkably, there is also a single more recent description of the structure [29], albeit without very detailed explanations of ontogeny, histochemistry, and ultrastructure.

To update our understanding of secretory sites in Hops, our study focused on investigating the origin, detailed structure (morphology, anatomy and ultrastructure), and distribution of the laticifer system, secretory idioblasts, and glandular trichomes throughout the plant body. We conducted analyses to determine the main classes of compounds present in latex and idioblast content in situ, aiming to identify their potential correspondence with important bioactive compounds. Additionally, we performed cytochemical tests to localize cellulase and pectinase activities, providing insights into the formation and growth of the laticifer system in this species.

## 2. Results

Three types of secretory structures constitute the secretory machinery of *H. lupulus*: laticifer system, secretory idioblasts and glandular trichomes. More detailed descriptions are provided for the laticifer and secretory idioblasts, given that secretory trichomes have been the subject of a greater number of anatomical and chemical studies.

### 2.1. Laticifer System

**Figure 1:**
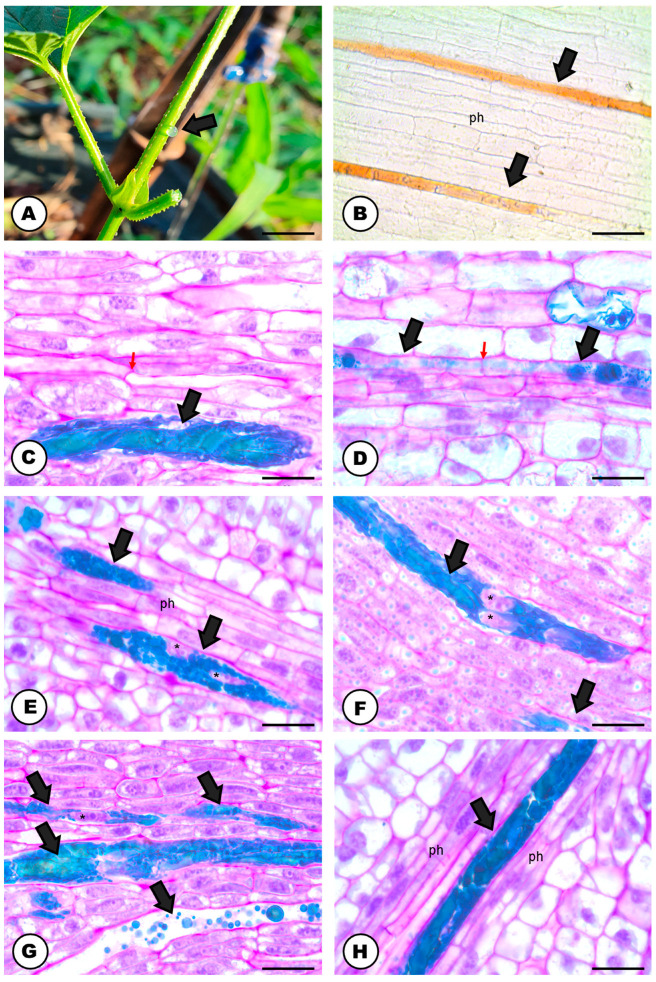
Laticifer system and latex of *Humulus lupulus*. (**A**) Colorless latex exuding from the internodal region of the stem. (**B**–**H**) Longitudinal sections. (**B**) Laticifer system (arrows) located between phloematic elements of a stainless section of petiole. Note the oxidized orangish color of the latex. (**C**,**D**) Precursor cells of the laticifer system in the apical portion of inflorescence buds. Note the terminal cell walls, showing the laticifer articulated structure (red arrows). (**E**,**F**) Laticifer system of pistillate flowers showing thicker cell walls (arrows) and nuclei (asterisks) with conspicuous nucleoli. (**G**,**H**) Laticifer system of pistillate flowers in detail showing the latex content stained with toluidine blue O (phenolics). Scale bars: (**A**) = 1 mm; (**B**) = 50 μm; (**C**–**H**) = 20 μm. Abbreviation: ph = phloem.

**Figure 2:**
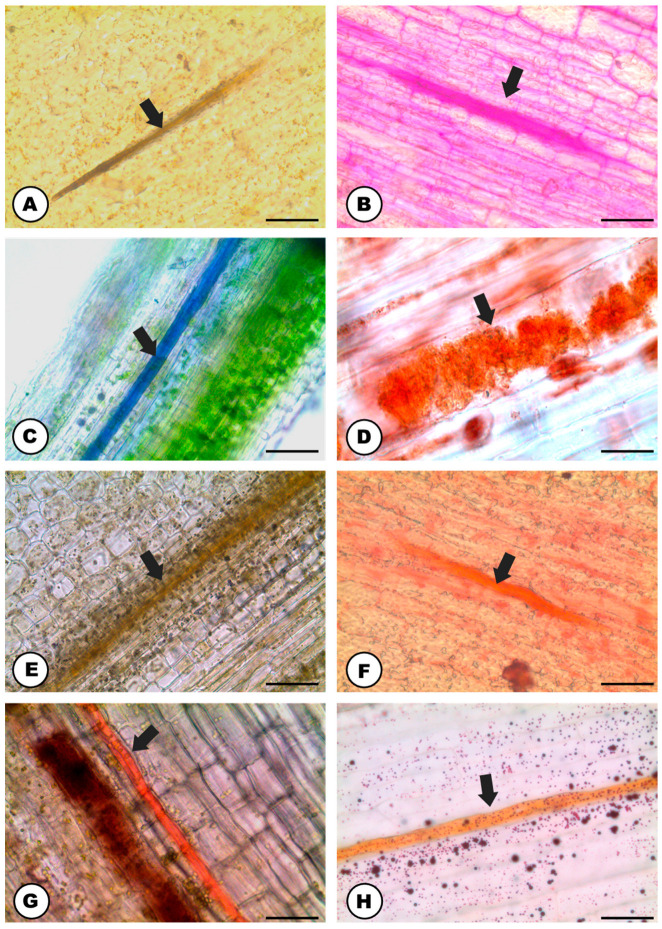
Histochemical analyses of the latex of the stem (**A**–**C**,**E**–**H**) and the petiole (**D**) of *Humulus lupulus* (longitudinal sections). Positive reaction of the latex (arrows) for: (**A**) Non-structural phenolic compounds (stain: ferric chloride). (**B**) Polysaccharides (stain: PAS reagent). (**C**) Terpenes (stain: Nadi reagent). (**D**) Rubber particles (stain: Oil O Red). (**E**) Alkaloids (stain: Wagner’s reagent). (**F**) Proteins (stain: xylidine Ponceau). (**G**) Tannins (stain: vanillin hydro chloridric acid). (**H**) Total lipids (stain: Sudan IV). Scale bars: (**A**–**C**,**F**,**G**) = 50 μm; (**D**,**H**) 20 μm; (**E**) = 100 μm.

The natural color of the latex is transparent (Figure 1A) and it becomes amber or orangish when in contact with the environment (Figure 1B). The latex contains phenolic compounds (Figure 1C–H and Figure 2A), polysaccharides (Figure 2B), terpenes (Figure 2C), rubber particles (Figure 2D), alkaloids (Figure 2E), protein bodies (Figure 2F), tannins (Figure 2G), total lipids (Figure 2H) and starch (Table 1). The latex is produced and stored in an articulated and unbranched laticifer system that traverses almost all analyzed organs of the plant (Figure 1), such as leaf, stem, pedicel, bracts, bracteoles, perianth, ovary and stigma. The exceptions are the anthers of the staminate flowers and the roots (Table 2).

The laticifer forms a system that originates from around three to five procambial cells close to the phloematic elements and articulations are formed by the dissolution of the terminal walls of precursor cells (Figure 1C,D). As the plant tissues develop, the laticifer grows among vascular elements (Figure 1C,D) or between other laticifer systems. This phenomenon allows laticifer system elongation by transforming precursor laticifer cells into one single tubular structure that is structurally linear, having no overall branches (Figure 1B–H and Figure 2A–H).

**Figure 3:**
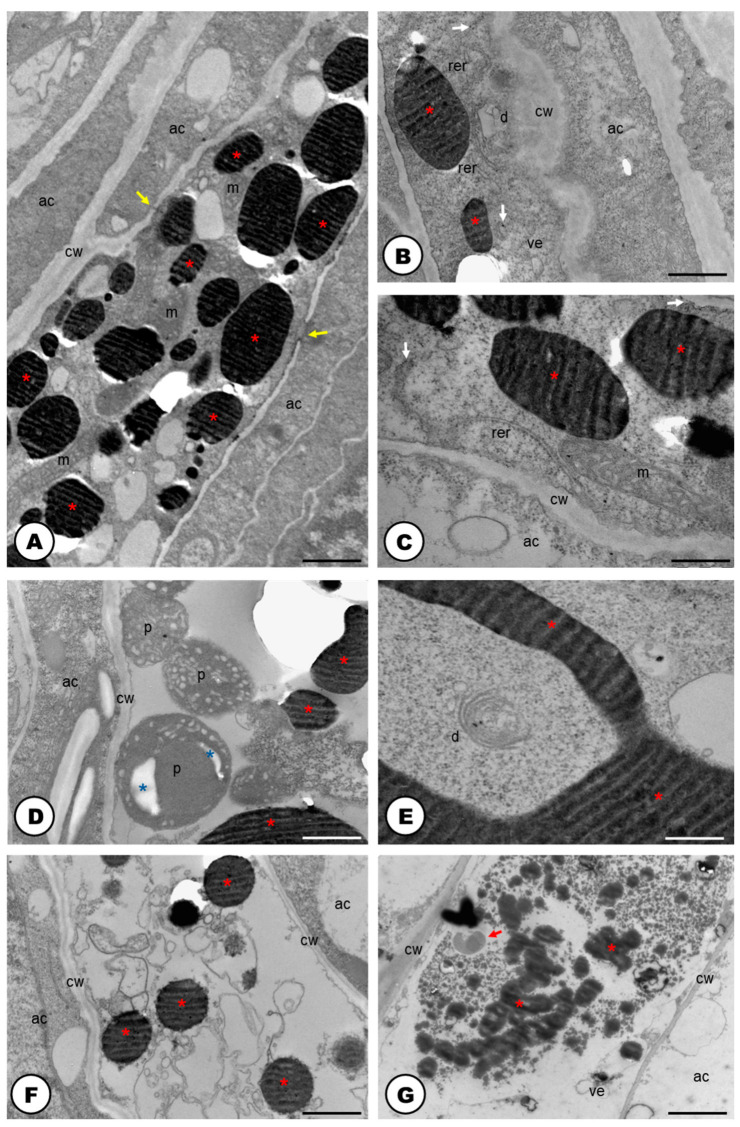
Ultrastructure of the cells forming the developing (**A**–**E**) and mature laticifer system (**F**,**G**) of *Humulus lupulus* (TEM). (**A**) Developing laticifer system with vacuoles filled with phenolics, mitochondria and vacuoles. Note that laticifer is connected with adjacent cells (yellow arrows). (**B**,**C**) Up-close view of the cytoplasm showing mitochondrion, rough endoplasmic reticulum, free ribosomes (white arrows), rough endoplasmic reticulum, dictyosomes with dilated vesicles and phenolic droplets (red asterisks). (**D**) View of the cytoplasm showing phenolic droplets (red asterisks) inside vacuoles and plastids with starch grains (blue asterisks). (**E**) Up-close view of the cytoplasm showing dictyosomes and phenolic droplets (red asterisks). (**F**) Up-close view of the cytoplasm showing organelles in an advanced stage of degradation and phenolic droplets (red asterisks). (**G**) Up-close view of the mature laticifer system cytoplasm full of phenolics (red asterisks), and secretion of unknown nature (red arrows). Scale bars: (**A**,**F**,**G**) = 3 μm; (**B**,**D**) = 1 μm, (**C**,**E**) = 500 nm. Abbreviations: ac = adjacent cell, cw = cell wall, d = dictyosome, m = mitochondria, p = plastid, rer = rough endoplasmic reticulum, ve = vesicle.

**Figure 4:**
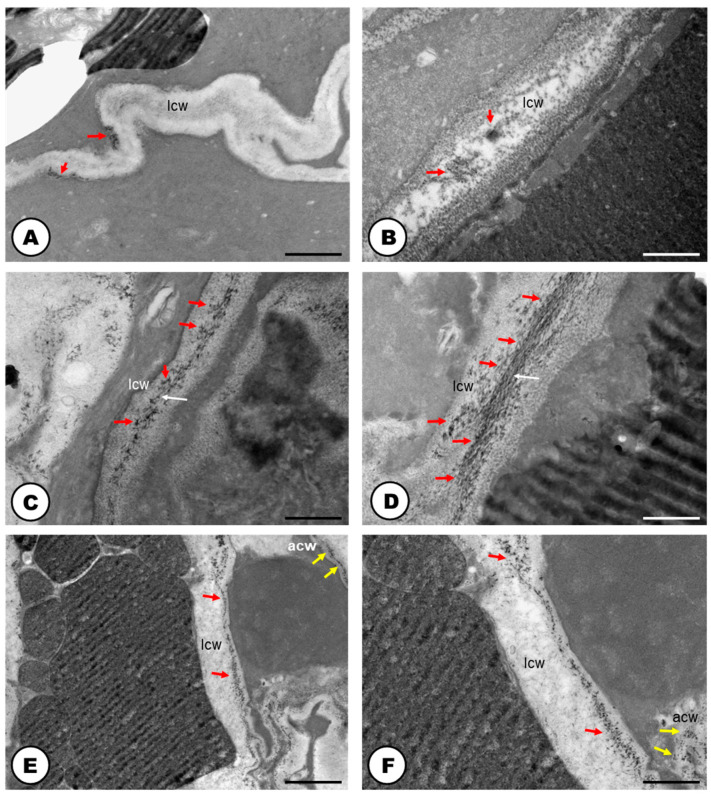
Cytochemistry of vegetative bud developing cells of the articulated laticifer system of *Humulus lupulus* (TEM). (**A**) Cellulase control sample, untreated with carboxymethylcellulose. The activity of the enzyme was positive (red arrows). (**B**) Pectinase control sample, showing positive reaction of the enzyme (red arrows). (**C**,**D**) Positive reactions for cellulase treated samples, showing cellulase activity in the cell wall close to the middle lamella (white arrows). (**E**,**F**) Positive reactions for pectinase treated samples. Enzyme activity was encountered in the cell wall. Note the electron-dense inclusions (red arrows) which are corresponding to the products of pectinase and cellulase activities in reaction with Benedict’s reagent. In the adjacent cells to the laticifer system, positive reactions for cellulase and pectinase were also observed but less intense (yellow arrows). Scale bars: (**A**,**F**) = 1 μm; (**B**–**D**) 500 nm; (**E**) 3 μm. Abbreviations: acw = adjacent cell wall, lcw = laticifer cell wall.

The cells forming the developing laticifer system possess oval nuclei (Figure 1E–G) and pecto-cellulosic walls that become thicker as they continue the development to a fully active secretory system (Figure 3A–D,F,G and Figure 4A–F). They show a cytoplasm that is gradually occupied by several phenolic droplets accumulated in vacuoles (Figure 3A–G), numerous mitochondria with conspicuous cristae (Figure 3A,C), dictyosomes with dilated vesicles (Figure 3B,E), free ribosomes (Figure 3B,C), extensive rough endoplasmic reticulum (Figure 3B,C), and plastids with starch grains (Figure 3D). The dictyosomes are formed of few cisterns and are usually located closely between the endoplasmic reticulum and the cell wall (Figure 3B,E). They produce exocytic and secretory vesicles, of the trans-Golgi network, which later are released into small vacuoles (Figure 3E). The small vacuoles formed contain latex substances (Figure 3A–E) and join to larger vacuoles in the end of the laticifer system development. The mature laticifer system is rich of vacuoles containing phenolics (Figure 3F,G), organelles in process of degradation (Figure 3F,G), and vesicles added to the remaining membranes (Figure 3G). Early in development, terminal cell walls are degraded by the combined action of cellulase and pectinase (Figure 4C–F), leading to the formation of a continuous system, with no nuclei and almost inconspicuous organelles.

In the cellulase control samples, untreated with carboxymethylcellulose (Figure 4A), the activity of the enzyme was also positive but less intense than in the treated samples (Figure 4C,D). Meanwhile, the positive reaction of pectinase was similar in both control (untreated with the enzyme) (Figure 4B), and the pectinase treated sample (Figure 4E,F). Positive reactions for cellulase (Figure 4A,C,D) and pectinase (Figure 4B,E,F) activities were found. Cellulase activity was encountered in the cell wall close to the middle lamella (Figure 4C,D). Positive reactions were also searched for vacuoles and endoplasmic reticulum, but no electron-dense crystalline inclusions were found. These electron-dense inclusions are corresponding to reducing sugars, which are the products of pectinase and cellulase activities in reaction with Benedict’s reagent. The reaction products can appear as widespread material (Figure 4B–F), or densely accumulated particles, which form groups in the cell wall (Figure 4A,B,D). In the adjacent cells to the laticifer system, positive reactions for cellulase and pectinase were also observed but they were less intense.

### 2.2. Secretory Idioblasts

**Figure 5:**
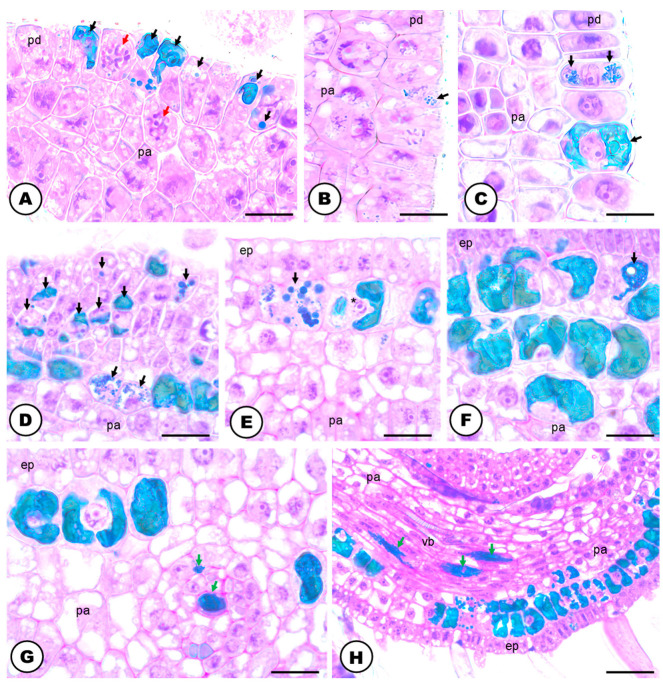
Developmental stages of phenolic idioblasts of *Humulus lupulus* (LM, stain: toluidine blue O). (**A**) Longitudinal section of a developing bracteole of a pistillate inflorescence showing precursor cells of phenolic idioblasts. Note the developing cells with mitotic figures (prophase—red arrows). (**B-D**) Longitudinal sections of developing bracteole (**B**,**C**) and bract (**D**) of a pistillate inflorescence, with developing idioblasts located in the protodermis, directly below it or near the developing parenchyma cells—note the blue droplets of secretion material slowly accumulating inside the vacuoles (black arrows). (**E**–**H**) Transversal sections of fully developed bracts of pistillate inflorescences, showing the differences in form and location of idioblasts and laticifer systems (green arrows). Scale bars: (**A**–**G**) = 20 μm; (**H**) = 50 μm. Abbreviations: ep = epidermis, pa = parenchyma, pd = protoderm, vb = vascular bundle.

**Figure 6:**
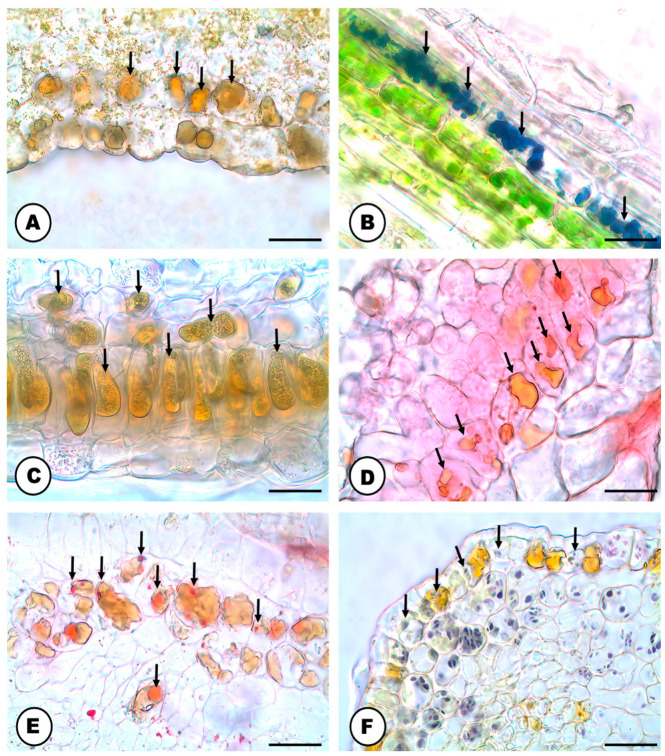
Histochemical analyses of the idioblast content (black arrows) of the idioblast cells in the leaf blade of *Humulus lupulus* (transversal sections). Positive reaction of the idioblast content for: (**A**) non-structural phenolic compounds (stain: ferric chloride). (**B**) Terpenes (stain: NADI reagent). (**C**) alkaloids (stain: Wagner’s reagent). (**D**) Proteins (stain: xylidine Ponceau). (**E**) Total lipids (stain: Sudan IV). (**F**) Starch (stain: Lugol’s solution) Scale bars: (**A**–**F**) = 20 μm.

**Figure 7:**
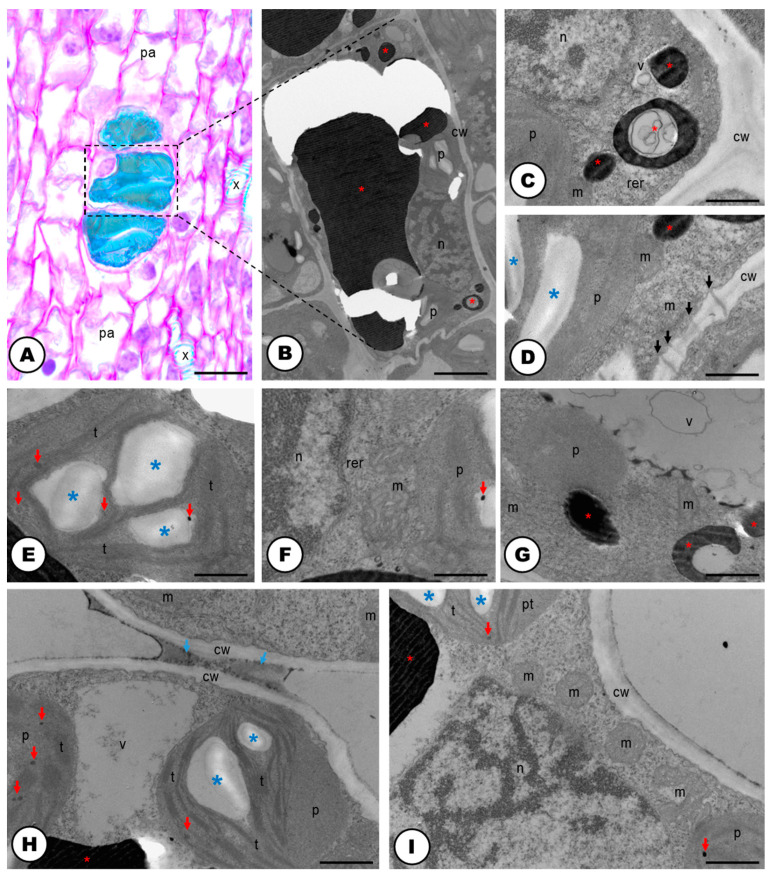
Anatomy (LM) and ultrastructure (TEM) of phenolic idioblasts of *Humulus lupulus*. (**A**) Longitudinal section of vegetative bud, with idioblast cells located near the differentiating parenchyma cells (stain: toluidine blue O) and xylematic cells. (**B**) Panoramic view of an idioblast cell of a vegetative bud with a thin cell wall, one large vacuole filled with phenolics (red asterisk), and an oval nucleus. (**C**) Up-close view of the cytoplasm, showing a portion of the nucleus, an amyloplast, a mitochondrion, a few small vacuoles and rough endoplasmic reticulum. (**D**) Up-close view of the cell wall between two idioblasts, highlighting the plasmodesmata (black arrows). (**E**) Up-close view of one amyloplast with stacked thylakoids, starch grains (blue asterisks) and plastoglobuli (red arrows). (**F**) Up-close view of the cytoplasm of an idioblast. Note a portion of the nucleus, a plastid with plastoglobuli, a mitochondrion and rough endoplasmic reticulum. (**G**) Up-close view of the vacuoles filled with phenolics. (**H**,**I**) Up-close view of the cytoplasm, plastids with starch grains and plastoglobuli, nucleus, numerous mitochondria and secretion between cell walls of two idioblasts (blue arrows, only in H). Scale bars: (**A**) = 20 μm; (**B**) = 2 μm; (**C**–**G**) = 500 nm; (**H**,**I**) = 1 μm. Abbreviations: cw = cell wall, m = mitochondria, n = nucleus, pa = differentiating parenchyma cell, p = neighboring plastids, rer = rough endoplasmic reticulum, t = thylakoids, v = vacuoles, x = xylem.

Similar to latex, the content of idioblasts also turns amber/orangish when oxidized. The idioblast secretion includes phenolic compounds (Figure 5A–H, Figure 6A and Figure 7A), terpenes (Figure 6B), alkaloids (Figure 6C), protein bodies (Figure 6D), total lipids (Figure 6E), starch grains (Figure 6F), polysaccharides and tannins (Table 1).

Phenolic idioblasts occur in all vegetative and floral organs of *H. lupulus* (Table 2), mainly in the subepidermal layers (Figure 5A–C,E–H).

They have pecto-cellulosic cell walls, and are mainly cube shaped but can also be amoeboid and even isodiametric (Figure 5A–H and Figure 7A,B), forming layers underneath the epidermis of the organs or being individually dispersed within the parenchyma (Figure 7A). The reproductive organs, mainly the bracteoles (Figure 5A–C) and bracts (Figure 5D–H), feature an enormous amount of idioblasts in their tissues in comparison with the vegetative organs, with the exception of the roots. 

The development of idioblasts begins during the differentiation of vegetative and reproductive organs. They can originate from cells of the protodermis and ground meristem (Figure 5A–C). These cells can go under mitosis, which allows the multiplication of a layer of idioblasts right under the epidermis. In these early stages, the meristematic cells are small in size and have a relatively dense cytoplasm that harbors small vacuoles and a round nucleus (Figure 5B–D). They are identified by the presence of multiple small sized vacuoles with phenolic droplets (Figure 5B–D). As development progresses, these small vacuoles merge, leading to the creation of one or a few prominent central vacuoles filled with phenolics that cover almost the entire cytoplasmic space (Figure 5D–H and Figure 7A,B). 

The fully developed idioblasts have thin cell walls composed of pectin and cellulose (Figure 5A–H and Figure 7A–E,G–I). The cells have one nucleus, which is very evident, usually spherical but can also be amoeboid (Figure 5A–G and Figure 7A–C,I). There is a single nucleolus, which is conspicuous in all developmental stages (Figure 5B–H and Figure 7C). The cytoplasm of the idioblasts exhibits amyloplasts (Figure 7D,E,H,I), rough endoplasmic reticulum (Figure 7C,D) and a high number of mitochondria (Figure 7C,D,F,G,I) close to the nucleus. The amyloplasts are often large and evident, with stacked thylakoids, sometimes even containing lipidic inclusions (Figure 7H,I). The idioblasts have plasmodesmata (Figure 7D) when in contact with each other.

### 2.3. Glandular Trichomes

**Figure 8:**
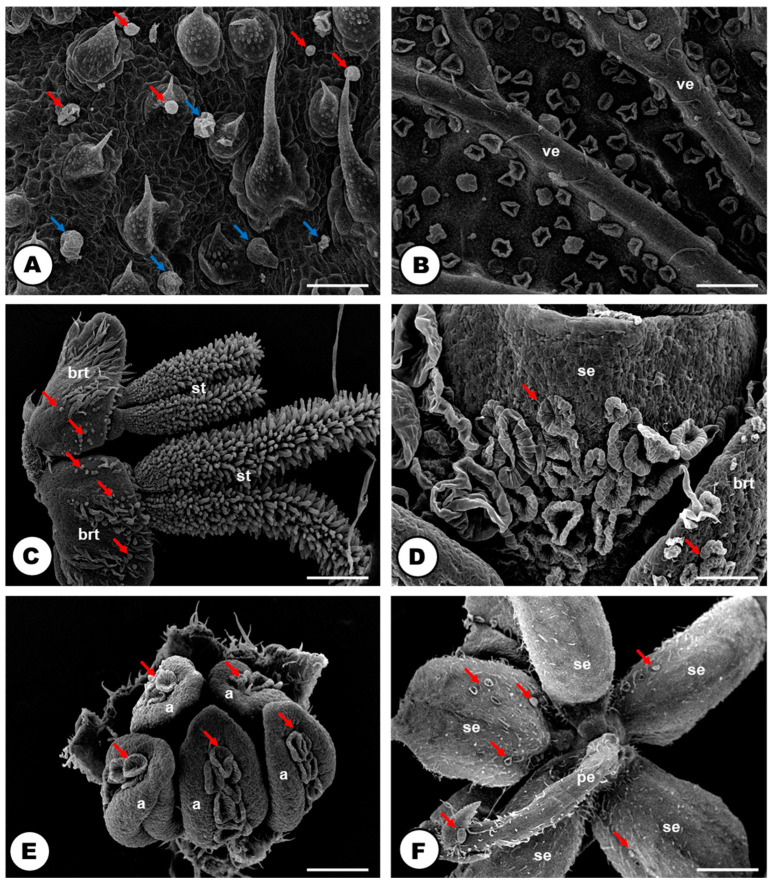
Distribution of glandular trichomes in the plant body of *Humulus lupulus* (SEM). (**A**) Capitate (blue arrows) and peltate trichomes (red arrows) on the adaxial side of the leaf. (**B**) Peltate trichomes on the abaxial side of the leaf. Note that the veins only possess tector (=non-glandular) trichomes in their surface. (**C**,**D**) Capitate and peltate trichomes in the pistillate flower. Note the abundance of glandular trichomes in the abaxial side of the sepal, and their absence in the stigma. (**E**,**F**) Capitate and peltate trichomes in the dorsal side of the anthers. (**E**) Adaxial side of the sepal (**F**) in a staminate flower. Note the abundance of glandular trichomes in the calyx, in the peduncle and only in the connective of the anthers. The glandular trichomes are pointed out by red arrows. Scale bars: (**A**,**D**,**E**,**F**) = 100 μm; (**B**,**C**) = 200 μm. Abbreviations: a = anther, brt = bracteole, pe = peduncle, se = sepal, st = stigma, ve = vein.

**Figure 9:**
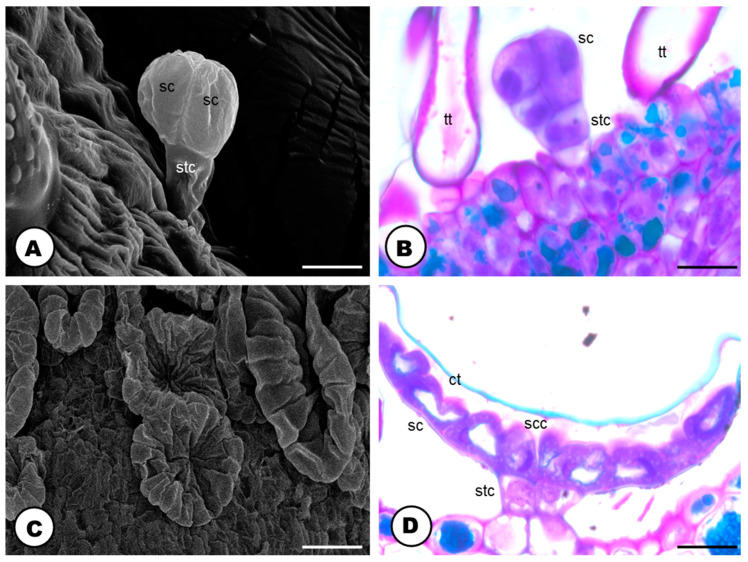
The structure of the capitate and peltate glandular trichomes located on the bracts of a pistillate flower of *Humulus lupulus* (**A**,**C**—SEM; **B**,**D**—LM, stained toluidine blue O). (**A**) Capitate trichome with a stalk and the secretory head cells. (**B**) Fully developed capitate glandular trichome. Note the elongate stalk cell and the round-shaped secretory head, formed by 3–4 cells with prominent nuclei. (**C**) Peltate trichomes without an exudate, thus, the flattened shape. (**D**) Fully developed peltate glandular trichome. Note the cuticle layer, the thinner blue-stained layer, that covers the interior region of the “folding” cells. Scale bars: (**A**,**C**) = 10 μm; (**B**,**D**) = 20 μm. Abbreviations: ct = cuticle, tt = tector trichomes (=non-glandular), sc = secretory cells, scc = subcuticular cavity, stc = stalk cells.

Both vegetative and floral parts of *H. lupulus* are covered with glandular trichomes (Figure 8 and Figure 9). They are widely present in the petiole and both adaxial (Figure 8A) and abaxial (Figure 8B) surfaces of the leaf blades, bracts and bracteoles. They were also observed in the sepal of the pistillate (Figure 8C,D) and staminate flowers (Figure 8E,F). They were not found on the stem, the root surface, the ovary and stigma in the pistillate flowers (Figure 8C) and the thecae in the staminate flowers (Figure 8E and Table 2). 

The glandular trichomes are numerous and robust, consisting of a multicellular stalk and head (Figure 8 and Figure 9). They can be found in two distinct morphotypes: capitate (bulbous) (Figure 8A and Figure 9A,B), and peltate (Figure 8 and Figure 9C,D).

The capitate trichomes are composed of a stalk with one to two cells and a rounded head with two to eight secretory cells (Figure 9A,B). The peltate ones are formed by a stalk with two to four cells and a cuticularized multicellular head, with the secretory cells radially arranged (Figure 9C,D); a large subcuticular space is formed in the secretory head when the released material is stored (Figure 8A,B,D,E and Figure 9C,D).

## 3. Discussion

Our data update the understanding of secretory sites in *Humulus lupulus* by providing details on the distribution of laticifer, phenolic idioblasts, and glandular trichomes along the plant body, the ultrastructure of secretory cells, and the chemical composition of the secretions. The ontogeny and ultrastructure of the laticifer system and secretory idioblasts, as well as the in-situ localization of substances in the latex and idioblast content, represent novel findings for the species. This study can contribute to a better comprehension of the ecological interactions between Hops and environmental factors, to the management of compound extraction on the plant body and to the understanding of the evolutionary history of secretory structures in Cannabaceae.

### 3.1. The Laticifer System in Humulus lupulus is Articulated, Unbranched and Crosses Almost All Organs of the Plant Body

The laticifer system of the Hop is here, for the first time, categorized as articulated and unbranched, considering (i) the presence of several precursor cells in the buds that will eventually compose the tube-like system; (ii) the observation of terminal cell walls in laticifer cells still in development, and the cellulase and pectinase activities in those walls close to the middle lamella; (iii) its linear structure with the absence of lateral projections or lateral anastomoses between close laticifers. Although our study is not the first report of the occurrence of a laticifer system in Hops [24,26,29], it configures not only a new extensive and detailed analyses of its morphology and ontogeny, but also an unprecedented work as it relates to its histochemistry, ontogeny, ultrastructure and cytochemistry.

Cellulase and pectinase act by dissolving cellulose and pectin in the terminal walls, thus contributing to the formation of the articulated laticifer system [41,42,43,44]. Thus, the information that *Humulus lupulus* exhibits a non-articulated laticifer system [24] cannot be corroborated. Such a misconception is probably due to the absence of ontogenetic and cytochemical analyses, in which it is possible to better interpret the formation and growth of the laticifer system.

The role of cellulase and pectinase has already been observed in species with articulated laticifers as we found in *H. lupulus*. These enzymes were detected in vacuoles of laticifers in *Ficus montana* and *Maclura tinctoria* (Moraceae) [44]; and in transverse cell walls of *Papaver somniferum* (Papaveraceae) [42]. The positive reactions for the two enzymes in these cell compartments would suggest that they are synthesized in the endoplasmic reticulum and are later translocated to the cell wall by exocytosis [44,45,46,47]. Additionally, the presence of a reaction product in the vacuoles indicates that endocytosis can occur as a means of the translocation of the products of the degraded cell wall to the vacuoles [48,49]. Surprisingly, the activity of these enzymes could not be identified in the vacuoles, endoplasmic reticulum, or transverse walls of Hop laticifer. The curious pectinase and cellulase activities found in the control tests can be explained by enzyme saturation: the addition of exogenous cellulose and pectin, along with the endogenous cellulase and pectinase in the middle lamella, does not alter the density of the reaction products in this region of the laticifer cells. Similar results were obtained for the articulated laticifer systems of Moraceae species [44].

The laticifer system is widely distributed through the plant body of *H. lupulus*, observed traversing the buds, the stem, petiole, and leaf blade, usually near the phloematic elements. There were also records of laticifers in the floral organs, except for the anthers in the staminate flowers. Such data is unprecedented for Hops, although it is in line with the data available in the literature for laticifer in general, which is known to accompany the vascular bundles along the various plant organs [1,50].

Current studies of Cannabaceae species (*Cannabis sativa* L., *Celtis pubescens* (Kunth) Spreng., *H. lupulus*, *Pteroceltis tatarinowii* Maxim. and *Trema micrantha* (L.) Blume) and even other Urticalean Rosids [51,52] showed laticifer systems in almost all the plant body including the leaf blade, petiole, stem, and floral organs. The differences in this system between the Urticalean Rosids are more related to the branches that may or may not occur. In *C. sativa*, the laticifer system is unbranched [52]; *Ficus* trees (Moraceae) show, in general, articulated and branched laticifer systems [51]; in Urticaceae a few studied species have unbranched laticifer systems [53,54,55], while others exhibit articulated and branched laticifers [44]. Thus, a recent hypothesis that proposes the presence of articulated and unbranched laticifer systems as a synapomorphy of Cannabaceae [52] is strengthened by this study.

### 3.2. The Latex of Humulus lupulus Seems to be Chemically Consistent to other Cannabaceae Species

By comparing the general classes of compounds detected in-situ in the latex of *Humulus lupulus* and other Cannabaceae species ([2,52], present study), a couple of differences are noted, such as the occurrence of phenolics only in the latex of *Cannabis sativa* and *H. lupulus*; and starch grains not being found only in *C. sativa*. Despite that, the main chemical classes of the latex composition in the family seems to be consistent, as protein bodies, total lipids and polysaccharides are found in all five Cannabaceae species studied so far ([52], present study) (Table 3).

It is noteworthy that the natural latex color is essentially the same in Cannabaceae species, being transparent in *H. lupulus* as well as *in C. sativa, C. pubescens, P. tatarinowii,* and *T. micrantha* [52]. However, the orangish color found for the latex of Hop after a longer contact with the environment constitutes a new observation within Cannabaceae. This variation in the oxidized color of the latex between *H. lupulus* and *C. sativa* (yellowish [52]) is possibly associated with minor differences in the latex metabolite composition, as it relates to specific chemical compounds that are included in the main classes detected by histochemistry. 

Also, the population of organelles found in the laticifer of *H. lupulus* seems to be strictly related to the chemical substances identified in its latex. Active dictyosomes are linked to polysaccharide production [1,56,57], and the extensive rough endoplasmic reticulum has a role in the phenolic production in the laticifer system [55,58,59,60], both of which were observed in this study. The presence of many mitochondria with conspicuous cristae in actively developing laticifer systems generates a significant amount of ATP synthesis and may also serve other metabolic processes carried out by this secretory structure [58,61].

The various secondary metabolites composing the latex and stored in vacuoles within the laticifer system are transported through fusion of vesicles derived from the endoplasmic reticulum, which also contributes to the growth of laticifer vacuoles. Interestingly, these vesicles from the endoplasmic reticulum can also fuse with the plasma membrane of laticifer, possibly containing pectinases and cellulases [44].

It is noteworthy that in *H. lupulus* distinct subcellular features were observed at different developmental stages of the laticifer system. Commonalities include a high concentration of phenolics in the latex, the presence of numerous vacuoles in developing and mature laticifer cells, an abundance of rough endoplasmic reticulum, ribosomes, and mitochondria in developing laticifer, and a lack of plasmodesmata in lateral cell walls. Notably, a developing laticifer possesses nuclei typically fusiform with a single nucleolus, while more a developed laticifer lacks endoplasmic reticulum, dictyosomes, and exhibits few peripheral organelles, possibly indicating cellular content autophagy [62], following latex secretion, as similarly seen throughout the development of the extrafloral nectary of *Citharexylum myrianthum* Cham. (Verbenaceae) [63].

The temporal variation in organelle populations, particularly the presence of abundant rough endoplasmic reticulum and dictyosomes in developing laticifers, is a feature seen in various plant species forming articulated [64,65] and non-articulated laticifer systems [66,67]. *H. lupulus*, however, lacks transverse walls even in some early laticifer developmental stages, likely due to the dissolution of these walls during first stages of laticifer development. Consequently, these walls are challenging to observe in TEM analyses, as pointed out by Marinho and Teixeira (2019) [44]. 

Furthermore, the extent of cytoplasmic component degradation differs between articulated and non-articulated laticifer systems. The distinct morphologies of laticifer systems differ in the extent of organelle degradation upon reaching maturity [44,67,68]. Autophagy is said to be involved in the developmental process of mature laticifers with mostly degraded cytoplasm and ruptured plasma membrane [67,69]. This observation aligns with the findings of this study, as fully developed laticifer cells of *H. lupulus* contain only latex products and a minimal peripheral cytoplasm. However, it is important to note that such subcellular machinery may not be applicable to all plant species, as some studies on articulated laticifer systems have shown diverse organelle compositions in their more developed stages, including the persistence of nuclei in specialized regions [44,60,70].

While the subcellular characteristics of laticifers can vary significantly, even within closely related groups with similar latex compositions [44,52], the presence of latex is a convergent trait that has independently evolved in various plant families [71]. Latex-bearing plants are diverse, spanning over 20,000 species across more than 40 different angiosperm families such as Asteraceae, Euphorbiaceae, Apocynaceae, Sapotaceae, and Moraceae [50,72,73]. Given this diversity in addition to the complexity and variation of latex chemical composition in different taxa, our understanding of the cytology of laticifer systems in the plant kingdom remains limited [51], and the subcellular population of the secretory cells in Hops can be updated and expanded with future research.

### 3.3. Phenolic-Secreting Cells of Humulus lupulus

The subcellular machinery of idioblasts found in Hops aligns with that of a phenolic-secreting cell, showing in general a high number of mitochondria next to rough endoplasmic reticulum, plastids with atypical morphology, and many plasmodesmata in cell walls [74]. Secretory idioblasts are classified based on the metabolites that their cytoplasm harbors at maturity. The literature recognizes a diverse range of secretory idioblasts, such as oil cells, phenolic cells, mucilage cells, tannin cells, crystal cells, and even latex cells [75]. 

The distribution of phenolic idioblasts in all plant organs of *H. lupulus*, primarily within the subepidermis of leaves and the stem parenchyma, along with the variety of morphological shapes, is also reported in other plant groups, such as ferns, gymnosperms, and angiosperms, including Apocynaceae, Euphorbiaceae, Fabaceae, Pinaceae, Primulaceae and Pteridaceae [75,76,77,78,79,80,81,82,83]. In closely related families to Cannabaceae, phenolic idioblasts can also be found in Ulmaceae, Moraceae, and Urticaceae [76]. The presence of phenolic idioblasts in all plant organs of *H. lupulus*, and a wide range of secondary metabolites in their secretion, indicate a probable important role in plant defense still not fully comprehended.

The origin and development of phenolic idioblasts in Hops from cells of the protodermis and the fundamental meristem, which multiply through mitosis, followed by cell vacuolation, align with the findings for tannin cells in two Fabaceae species (*Dimorphandra mollis* Benth. and *Stryphnodendron adstringens* (Mart.) Coville) [74]. The authors of this study also discuss that the development of idioblasts involves the process of vacuolation of meristematic cells [74]. The physiological factors which induce the development and the serial processes that lead to the formation of idioblasts in plant tissues is still not fully understood. It is suggested that cellular differentiation or specialization can arise from the partitioning of distinct biochemical systems within a single parent cell, and this segregation becomes permanent through the formation of cell walls between the two sister cells [75,84]. Future studies with Hops may reveal the influence of environmental and physiological factors in the development, distribution and chemical content composition of secretory idioblasts.

The widespread distribution of phenolics in both the laticifer system and secretory idioblasts of Hops can lead to misidentification between these two types of secretory structures. Additionally, they possess similar dimensions in anatomical sections, along with pecto-cellulosic cell walls and very similar cytoplasmic content, especially a high concentration of phenolics. This confusion may have delayed and even prevented detailed studies of the laticifers and other secretory structures in Hops.

### 3.4. Humulus lupulus Show an Extensive Cover of Glandular Trichomes 

The first line of defense in Hops and other Cannabaceae species appears to be at the plant surface: the substances produced by the capitate trichomes come into direct contact with the environment since this morphotype of glandular trichome exhibits a very thin cuticle and the exudate is not stored in a subcuticular space. Thus, the metabolites produced are directly released into the trichome surface. On the other hand, in the peltate glandular trichomes, the exudate is stored in a wide subcuticular space, and is only released to the environment after cuticle breakage by external factors, such as herbivores, for example [85]. Such defense should protect against excessive UVB radiation, higher temperatures, and damage caused by herbivorous insects or infectious diseases [1,65,85].

Glandular trichomes have undergone extensive study within Cannabaceae, especially in *C. sativa* [2,4,25,39,86] and *H. lupulus* [30,32,33,34,35,36,37,38,87]*,* with some reports for the genera *Trema*, *Celtis* and *Pteroceltis* [88]. The prevalence of glandular trichomes in various parts of the plant body, particularly in wind-pollinated species as Hops and other Urticalean Rosids [88,89,90], suggests their primary role in defense against herbivory [85,91] rather than attracting pollinators. The defensive function of glandular trichomes against herbivory has been previously documented in *C. sativa* [92] and even *H. lupulus* [93], where the action of chemical compounds secreted by trichomes is ‘triggered’ when herbivores, especially caterpillars, disturb peltate trichome head cells, leading to the release of trichome exudate.

The capitate and peltate types of glandular trichomes, widely reported for the Hops [35,36,37,38], were found covering all the exposed plant body including floral parts, except the stem and the thecae of staminate flowers. The occurrence of glandular trichomes in almost all parts of staminate inflorescences, such as bract, bracteoles, peduncle, calyx, filament and anther connective, is a new report for the species. The lack of studies on staminate inflorescences could be related to the research focus on the peltate glandular trichomes of pistillate flowers, which are extensively researched due to their main role in beer production and potential pharmacological properties [8,9,30,32,33,34,35,36,37,38,39,94,95,96]. 

Over the past few decades, it has been widely disseminated that the Hop glandular trichomes (lupulin glands) of pistillate inflorescences are the primary contributors to the bitter taste and characteristic aroma of beer [8,9]. Hop bitter resins and essential oils, which give beer its organoleptic properties, accumulate mainly in the peltate glandular trichomes [8], which is corroborated by studies on metabolic pathways of essential oils [8,87] and resins [97].

## 4. Materials and Methods

Samples of vegetative buds, young and fully expanded leaves, young stems (third and fourth internodes), young roots, pistillate and staminate flowers were collected from specimens of *H. lupulus* cultivar *Cascade* (USA) cultivated in the greenhouse of the Medicinal Garden of FCFRP/USP and the Center of Studies in Olericulture and Improvement (NEOM), UNESP, Jaboticabal campus. The voucher for the individuals of Hops cultivated in the Medicinal Garden was deposited in the SPFR herbarium (FFCLRP/USP) (access number 17782). 

For anatomical analyses, the samples were fixed for 48 h [98] in buffered formalin [99], dehydrated in an ethanolic series, embedded in histological resin (Historesin, Leica, Wetzlar, Germany) and sectioned in longitudinal planes (5 μm), using a rotary microtome (Leica RM 2245). The sections were stained with 0.1% toluidine blue O in phosphate buffer, pH 6.8 [100], and examined under a light microscope. For in situ detection of compounds, two anatomical slides of each plant organ (roots, stem, leaf, pistillate and staminate inflorescences) were stained with each staining reagent. Positive reaction was considered when obtained in both anatomical slides of each organ. The following reagents were used: toluidine blue O for phenolic compounds and mucilage [100], xylidine Ponceau for protein bodies [101], periodic acid-Schiff (PAS) for polysaccharides [102] and Sudan IV for total lipids [103]. Reference-material (sections untreated with stain reagents) were used as controls for toluidine blue O and Sudan IV [100,101] reagents; control sections for xylidine Ponceau staining were treated with 10% acetic anhydride in pyridine for 2–4 h [101]; and PAS control sections were untreated with periodic acid [102]. Free-hand sectioning of stem, petiole, and leaf was also performed, and tested using the following reagents: Wagner for alkaloids [2], Nadi for terpenes and essential oils [104], Oil O Red for rubber particles [105], ferric chloride for non-structural phenolic compounds [98], vanillin hydrochloric acid for tannins [106] and Lugol for starch grains [98]. Reference-material untreated with stain reagents were used as control for all free-hand sections [107]. A Leica DFC 295 digital camera was used in conjunction with a Leica DM 5000 B light microscope to take photomicrographs.

For surface examination of glandular trichomes, young leaves, pistillate and staminate flowers were transferred to 80% ethanol and placed in 90% ethanol inside a micropore for 1 h. Then, it was transferred to 100% ethanol and brought to the critical point with CO_2_. The material was built on stubs and metallized with gold for observation in a scanning electron microscope Zeiss EVO-50 (Cambridge, UK) located at FMRP/USP. The morphological classification of the glandular trichomes was performed according to references [8,35,36], some of the few existing works in the literature that emphasize the detailed morphology and ontogeny of this secretory structure in *H. lupulus*. 

For ultrastructural analysis of the laticifer system, idioblasts, and glandular trichomes, small samples of young stem and petiole, and vegetative buds were collected and fixed in Karnovsky’s solution for 24 h [108] and post-fixed in 1% osmium tetroxide in 0.1 M phosphate buffer (pH 7.2). Then, the samples were washed in distilled water, dehydrated, and embedded in Araldite (EMS 6005). The sections were cut using a Leica Reichert Ultracut S ultramicrotome at 60–70 nm. They were collected on copper grids and contrasted with 2% uranyl acetate [109] and lead citrate [110] for 15 min. Using a Jeol 100CX II instrument (JEOL Ltd., Akishima, Tokyo), transmission electron micrographs were obtained.

Cytochemical localization at the ultrastructural level of cellulase and pectinase activities was performed to check the laticifer formation. The vegetative buds were collected, fixed in Karnovsky’s solution for 24 h [108], washed 10 times in 0.1 M phosphate buffer (pH 7.2) and maintained in the buffer at 4 °C for the night. For cellulase activity test, the samples were incubated in 0.05 M citrate buffer (pH 4.8), for 10 min at room temperature, with 0.02% carboxymethylcellulose [111,112]. For pectinase activity analysis, the bud samples were incubated in 0.1 M sodium acetate buffer (pH 5.0), for 20 min at room temperature, with 0.5% pectin [113]. Control samples were incubated in the same buffers as the treatment samples, but they were not exposed to the enzymes. Treatment and control samples were moved to Benedict’s reagent and heated to 80 °C for 10 min, washed in 0.1 M phosphate buffer, and post-fixed in 1% osmium tetroxide for 2 h [112]. The samples were then further processed using the usual methods of ultrastructural analysis.

## 5. Conclusions

This study provides new and unprecedented information about the secretory machinery of Hops: (i) detailed distribution, morphology, ontogeny, histochemistry, ultrastructure and cytochemistry of the laticifer system; (ii) distribution, ontogeny, histochemistry and ultrastructure of phenolic idioblasts, and (iii) occurrence of glandular trichomes in particular organs of the staminate inflorescence.

The laticifer system, phenolic idioblasts and glandular trichomes may compose an important protective glandular system of the ovary and ovules in pistillate flowers of *H. lupulus*. However, the precise functions of latex and idioblast secretion in the gynoecium, especially regarding wind pollination, require further investigation. The wide distribution of laticifers and phenolic idioblasts in the reproductive organs of Cannabaceae [52] suggests a significant protective function for these secretory structures in safeguarding the family flowers from predators and pathogens.

The progress in research on elucidating the exudate of glandular trichomes, the chemical composition of the latex and the secretion of phenolic idioblasts has the potential to broaden their applications in food and pharmaceutical industry, and enhance our understanding of their roles in plant-environment interactions.

## Figures and Tables

**Table 1:** Histochemical analyses performed in the latex and idioblast content of *Humulus lupulus*. Symbols: (+): present; (−): absent.

Reagents	Target Compound	Latex	Idioblast Content
Toluidine blue O (purple)	Mucilage	−	−
Toluidine blue O (green)	Phenolics	+	+
Xylidine Ponceau	Protein bodies	+	+
Sudan IV	Total lipids	+	+
PAS reagent	Polysaccharides	+	+
Wagner’s reagent	Alkaloids	+	+
Nadi reagent	Terpenes	+	+
Oil O Red	Rubber particles	+	−
Ferric chloride	Non-structural phenolics	+	+
Vanillin hydrochloric acid	Tannins	+	−
Lugol’s solution	Starch	+	+

**Table 2:** Distribution of the secretory structures of *Humulus lupulus* in vegetative and reproductive organs. No difference in the distribution of the two morphotypes of the glandular trichomes (capitate and peltate) was observed. Symbols: (+): present; (−): absent; (empty area): not applicable.

Plant Organ	Laticifer System	Glandular Trichomes	Phenolic Idioblasts
Stem		−	
Cortex	+		−
Medula	−		−
Petiole	+	+	+
Leaf (adaxial side)			
Leaf blade	−	+	+
Central vein	+	+	−
Parallel veins	+	+	+
Leaf (abaxial side)			
Leaf blade	−	+	+
Central vein	+	−	−
Parallel veins	+	−	+
Staminate inflorescence			
Peduncle	+	+	+
Bracts	+	+	+
Perianth	+	+	+
Filament	−	+	+
Anthers			
Connective	−	+	+
Thecae	−	−	+
Pistillate inflorescence			
Peduncle	+	+	+
Bracts	+	+	+
Sepals	+	+	+
Ovary	−	−	+
Stigma	−	−	+
Root		−	
Cortex	−		+
Medula	−		+

**Table 3:** Latex composition in some Cannabaceae species. The data for *H. lupulus* were obtained in the present study, and for other species, they were obtained from Leme et al. [52]. Symbols: (+) present; (−) absent; (?) not analyzed.

Target Compounds	*Humulus* *lupulus*	*Cannabis* *sativa*	*Celtis* *pubescens*	*Pteroceltis* *tatarinowii*	*Trema* *micrantha*
Phenolics	+	+	−	−	−
Non-structural phenolics	+	+	−	−	−
Tannins	−	?	−	?	−
Proteins	+	+	+	+	+
Total lipids	+	+	+	+	+
Terpenes	+	?	+	?	+
Rubber particles	+	?	?	?	?
Polysaccharides	+	+	+	+	+
Starch	+	−	+	+	+
Alkaloids	+	?	−	−	−

## Data Availability

Data are contained within the article.
